# The competitive strategies of poisonous weeds *Elsholtzia densa* Benth. on the Qinghai Tibet Plateau: Allelopathy and improving soil environment

**DOI:** 10.3389/fpls.2023.1124139

**Published:** 2023-03-06

**Authors:** Xijie Zhou, Yunxing Xiao, Danwei Ma, Yusi Xie, Yu Wang, Hong Zhang, Yanan Wang

**Affiliations:** College of Life Science, Sichuan Normal University, Chengdu, China

**Keywords:** *Elsholtzia densa* Benth., competitive strategies, allelopathy, root border cells, soil environment

## Abstract

**Introduction:**

The competitive strategies of plants play a crucial role in their growth. Allelopathy is one of the weapons that plants use to improve their competitive advantage.

**Methods:**

In order to explore the competitive strategy of a poisonous weed *Elsholtzia densa* Benth. (*E. densa*) on the Qinghai-Tibet Plateau (QTP), the effects of decomposing substances of *E. densa* on growth, root border cells (RBCs) characteristics of highland crop highland barley (*Hordeum vulgare* L.), and soil environment were determined.

**Results:**

The decomposing allelopathic effect of *E. densa* on the germination and seedling growth of highland barley mainly occurred in the early stage of decomposing. The allelopathic effects were mainly on seed germination and root growth of highland barley. After treatment with its decomposing solution, the RBC’s mucilage layer of highland barley thickened, and the RBC’s activity decreased or even apoptosis compared with the control. However, only the above-ground part of the treatment group showed a significant difference. The effects of *E. densa* decomposed substances on the soil environment were evaluated from soil physicochemical properties and bacterial community. The results showed that soil bacteria varied greatly in the early stage of decomposion under different concentrations of *E. densa*. In addition, *E. densa* decomposing substances increased the soil nutrient content, extracellular enzyme activities, and bacterial community diversity. In the process of decomposition, the bacterial community structure changed constantly, but Actinobacteriota was always the dominant phylum.

**Discussion:**

These results indicated that *E. densa* might adopt the following two strategies to help it gain an advantage in the competition: 1. Release allelochemicals that interfere with the defense function of surrounding plants and directly inhibit the growth and development of surrounding plants. 2. By changing the physical and chemical properties of soil and extracellular enzyme activity, residual plant decomposition can stimulate soil microbial activity, improve soil nutrition status, and create a more suitable soil environment for growth.

## 1 Introduction

As one of the most sensitive regions in the world to climate change, the Qinghai-Tibet Plateau (QTP) has great ecological fragility (Peng et al., 2012). In recent decades, a combination of adverse factors has led to serious land degradation on the QTP, resulting in a sharp decline in biodiversity and productivity (Geissler et al., 2019). Land degradation also changes the organization of plant communities, and poisonous weeds that are more tolerant to harsh environments take advantage of this opportunity to expand (Peng et al., 2020), gradually replacing native crops as new dominant species. At the same time, soil resources, microbial communities, and the spatial pattern of other groups changed dramatically (Dong et al., 2020).

Previous reports have shown that poisonous weeds compete with surrounding plants for natural resources such as light, water, and nutrients (Mushtaq et al., 2019) and release allelochemicals into the soil, like the “Novel weapon” hypothesis of invasive plants, directly inhibiting the growth of other plants in the habitat. They also disrupt the interaction between underground beneficial microbes and surrounding plants, thereby indirectly interfering with the growth and development of surrounding plants (Kalisz et al., 2020; Qu et al., 2021).

Allelochemicals can be released into the environment through various ways, such as volatilization, rain and fog leaching, root secretion, and decomposition of plant residues or litters (Hickman et al., 2021; Nikolaeva et al., 2021). After the allelochemicals enter the environment, they interact with the environment through retention, transformation, and migration, eventually leading to the increase or decrease of allelopathy (Xiao et al., 2017). Compared with hydrophilic allelochemicals, lipophilic allelochemicals may stay longer and continuously affect the surrounding plants through soil retention (Whitehead et al., 2018).

During evolution and in response to external stresses, including allelochemicals, root tips that were originally exposed directly to soil generated and released root border cells (RBCs) (Ropitaux et al., 2020). The RBCs and the mucus secreted by the plant themselves form a protective sheath of the root tip, which builds a biological, chemical, and physical barrier between the plant-root-external environment. The RBCs can attract and capture soil organisms and microorganisms, adsorb and neutralize the soil’s toxic substances and regulate the rhizosphere environment (Carreras et al., 2020). Therefore, the allelochemicals entering the soil must break through the biological defense line constructed by the RBCs to affect the growth and development of the root system.

In the microenvironment comprising plant root-soil-microbe interactions, the soil microbes are the bridge connecting plants to soil and plants to plants. They can decompose complex components in the soil to form small molecular substances, helping the plant roots to absorb nutrients and improve stress resistance (Zhang et al., 2020). However, the allelochemicals released by plants disrupt the balance between the surrounding plants and soil and establish new microbial communities mutually beneficial to themselves (Li et al., 2022). Soil bacteria influence potential soil functions (nutrient cycling and climate regulation) more than soil fungi in a highly cold environment (Wang et al., 2021).

The plant community affected by poisonous weeds will gradually change from perennial herbs (gramineaceae and sedge family) to annual herbs and eventually form a single dominant community of poisonous weeds. During the evolution process, soil moisture and fertility decreased gradually, and community species composition and the carbon water cycle in the ecosystem were seriously affected (Qin et al., 2022). *Elsholtzia densa* Benth. (*E. densa*) is a dominant poisonous weed widely distributed in the QTP (Duan et al., 2019), which is a serious hazard to the farmland ecosystem of the QTP (Shang et al., 2018; Xiao et al., 2021). As an annual herb, *E. densa* produces manydead twigs and withered leavesyearly, and its decomposition inevitably releases allelochemicals into the soil continuously. Therefore, we have two hypotheses: 1) *E. densa* might interfere with the defense function of surrounding plants and directly inhibits the growth and development of surrounding plants by releasing allelochemicals; 2) *E. densa* may change the soil environment by residual plant decomposition.

In order to verify the above hypothesis, this study used the highland barley (*Hordeum vulgare* L.), a gramineous crop with strong adaptability to the high cold environment of the QTP (Li et al., 2020), as the receptor. The seeds, seedlings, and RBCs of highland barley were treated with different decomposing parts of the decomposing solution. These results were used to evaluate the allelopathic potential of *E. densa* decomposing solution and its effect on defense indexes of RBCs. In addition, different contents of *E. densa* residue were mixed in the soil and divided into short and long time stages of decomposition. Under decomposition time and residue concentration, two variables were studied: the change law of nutrient content, extracellular enzyme activity, and bacterial diversity of decomposition soil.

## 2 Materials and methods

### 2.1 Materials and sources

The plants of *E. densa* and soil are collected at an altitude of 3470 m from Axi Township (102°55.944′ E, 33°41.023′ N), Hongyuan County, Aba Tibetan, and Qiang Autonomous Prefecture, Sichuan Province, China. The collected *E. densa* plants were at the maturity stage. The entire plant was divided into roots and aerial parts, dried in the shade under natural conditions, and cut into small sections of about 2 cm. In order to avoid interference, soil without *E. densa* growth in the area was selected for the test. We scooped up the topsoil (depth 0-20 cm) with a sampling shovel and placed it in a sealed bag. The soil samples were returned to the laboratory and sieved into 1 mm for use. The highland barley seeds were purchased from Daofu County, Garze Tibetan Autonomous Prefecture.

### 2.2 Allelopathic effect and cytotoxicity experiments of decomposing solution

#### 2.2.1 Preparation of decomposing solution

The root decomposing solution (RD) and the above-ground decomposing solution (AD) were prepared according to the biomass of *E. densa* residues. RD = 25 groot: 500 g soil: 1 L water, AD = 50 g of aerial parts of *E. densa*: 500 g soil: 1 L water. The above components were evenly stirred and sealed at room temperature to decompose away from light. The decomposition process was divided into short-term 30 days (d) and long-term 60 d, during which the mixture was stirred once every 7 d, and each treatment was repeated 3 times. At the end of decomposition, clean gauze was used to remove the coarse substances in the decomposition solution, and the vacuum pump was used to extract the remaining filtrate under the filter paper. Then the filtrate volume after extraction was fixed to 1 L to obtain raw RD (25 mg/mL) and raw AD (50 mg/mL), respectively. During the experimental treatment, both were diluted with distilled water to obtain different concentrations of treatment solutions. The RD concentrations were 5, 10, 15, 20, and 25 mg/mL, and the AD concentrations were 10, 20, 30, 40, and 50 mg/mL.

#### 2.2.2 Seed germination test

The highland barley seeds with full grains and no pest damage were sterilized with 0.5% KMnO_4_ solution for 15 minutes (min) and then rinsed repeatedly with distilled water. After soaking in a 25°C incubator for 4 hours (h), the seeds were spread evenly in a germination box (12 cm × 12 cm × 9 cm) padded with two layers of filter paper, with 60 seeds per box. 15 mL of different concentrations of RD and AD were added to the germination box, and 15 mL of distilled water was used as the control. The number of germinated seeds and rooting was counted every day in a 25°C incubator for 7 d. The temperature in the incubator was set to 25°C/20°C, the illumination period to 12 h/12 h (light/dark), and the illumination to 2000 lx. The germinated germ reached half the length of the seed as germination, and the radicle reached half the length of the seed as rooted. Next, we calculated the following parameters (Sun et al., 2021):


$$\begin{array}{c} GR=\frac{GN}{SN}\times 100\%\\ GI=\Sigma (\frac{G_{t}}{D_{t}})\\ RR=\frac{RN}{SN}\times 100\% \end{array}$$

where *GR* is the germination rate, *GN* is the number of germinated seeds, *SN* is the total number of seeds, *GI* is the germination index, *Gt* is the germination number on day *t*, *Dt* is the corresponding germination day, *RR* is the rooting rate, and *RN* is the number of rooted seeds.

#### 2.2.3 Seedling growth test

The highland barley seeds were disinfected according to the steps in 2.2.2 and then immersed in the incubator at 25°C for 12 h. The seeds, after soaking, were transferred to a porcelain plate covered with wet double sterile gauze and continued to promote germination without light for 24 h. After the seeds were exposed to the white root tips, the full seeds with the same radicle lengthwere selected and inserted into a small pot covered with 3 cm perlite (diameter 10 cm, height 6 cm, 300 g perlite). Then we added 50 mL 1/4 MS nutrient solution and poured the nutrient solution every 1 day. The small flower pots were cultured in a light incubator for 4 days with a light intensity of 4500 lx, a light cycle of 12 h/12 h (light/dark), and a diurnal temperature of 25°C. After the first true leaves of the highland barley seedlings grow, 20 mL of different concentrations of RD and AD were added and distilled, using water as a control. Each treatment was repeated three times. After adding the decomposing solution, the seedlings were cultured for 7 d. After the cultivation, the seedlings werewashed with distilled and dried with filter paper. The plant height and root length of the highland barley seedlings were measured. After defoliating at 105°C and drying to constant weight at 85°C, the dry weight of the seedlings and roots wasmeasured (Sun et al., 2021).

#### 2.2.4 Toxicity test of a decomposing solution to RBCs

The radicles of the highland barley seeds with exposed white root tips are inserted into a culture flask (volume 200 mL, diameter 9 cm, height 8.5 cm) containing 0.8% pure agar medium and cultured upside down. When the root length reaches 40 mm (at this time, the number of RBCs reaches the peak, and the activity is high), 10 root tips with a length of about 5 mm are randomly selected and placed in 100 μL of 25 mg/mL RD and 50 mg/mL AD treatment solutions vortexed for 30 seconds (s). Then we rinsed the root tip twice with 50 μL of the treatment solution to prepare a cell suspension (200 μL), which was mixed and placed in an incubator at 25°C for 30 min in the dark. Each treatment was repeated six times.

Referring to Singh’s method, 20 μL of cell suspension was added to a centrifuge tube (0.5 mL capacity), and 8 μL of AO/EB mixed dye solution was added dropwise and stained for 2 min. The cell viability and morphology were observed under a LEICA DM300 fluorescence microscope, where the orange ones are dead cells, and the green ones are living cells (Singh et al., 2021). Each treatment was repeated five times. Then we calculated the cell viability:


RBCs survival rate=number of viable cells/total number of cells×100%

Referring to the method of Ropitaux, 8 μL of the cell suspension was taken, and 8 μL of Indian ink was added. After being stained for 10 min, the cells were observed with a Nikon ECLIPSE 55i microscope at x40 in a bright field, and pictures were captured to measure the thickness of the mucilage layer (Ropitaux et al., 2020). Each treatment measured 10 cells, and each cell measured 6 different locations.

### 2.3 Determination of soil properties

#### 2.3.1 Experimental treatment

We pulverized the dried plants of *E. densa* with a crusher and prepared the decomposed soil according to the residue content of *E. densa* in 0, 0.02, 0.04 g/g, and 0.08 g/g, and added an appropriate amount of distilled water to keep the soil moist. In order to have sufficient samples to repeat the test, the total amount of soil in each treatment group was 300 g. Each treatment was repeated three times, and the soil samples were collected on 20 d, 40 d, and 60 d, respectively, some of which were tested for soil nutrients and enzyme activities, while the others were stored in a -80°C refrigerator to determine bacterial diversity.

#### 2.3.2 Soil nutrient determination

The determination method was slightly modified by referring to Pan et al. (Pan et al., 2016; Geng et al., 2017; Li et al., 2021). The soil’s pH was measured based on the electrode potential method (air-dried soil: distilled water = 1:2.5). The soil organic matter (SOM) was determined by the potassium dichromate volumetric method. In contrast, the total phosphorus (TP) and the available phosphorus (AP) were determined using the sodium hydroxide alkali fusion-molybdenum antimony anti-colorimetric method. The absorbance values at 585 nm, 700 nm, and 882 nm were measured using a SpectraMax M2 microplate reader (Molecular Devices, USA). Both the total potassium (TK) and available potassium (AK) were measured using a Z-2000 Zeeman atomic absorption spectrophotometer and the ammonium acetate extraction-flame photometer method. The total nitrogen (TN) and available nitrogen (AN) were determined by an automatic Kjeldahl analyzer (FOSS, KJELTECTM 2300).

#### 2.3.3 Soil enzyme activity determination

The determination method was slightly modified by referring to Xie et al. (Chen et al., 2020; Xie et al., 2020). The urease activity was determined by the sodium phenolate colorimetric method, and the invertase and cellulase activities were determined by the 3,5-dinitrosalicylic acid colorimetric method. The polyphenol oxidase activity was determined by the colorimetric method for measuring pyrogallol, while the acid phosphatase activity was measured using the Solarbio soil acid phosphatase (S-ACP) activity assay kit.

#### 2.3.4 High throughput sequencing of bacteria

The genomic DNA of the soil samples was extracted using the DNA extraction kit to detect the DNA concentration and purity. By employing the diluted genomic DNA as a template, the V3-V4 region of the bacterial 16S DNA was amplified by PCR using specific primers with Barcode and the Takara Ex Taq high-fidelity enzyme (Takara, Japan). The primer sequences were the 343 F (5’-TACGGRAGGCAGCAG-3’) and 798 R (5’-AGGGTATCTAATCCT-3’). After performing electrophoresis detection on the PCR product and purifying it using magnetic beads as a template for two rounds of PCR amplification, the second-round PCR products were electrophoresed again and purified with magnetic beads for Qubit quantification. An equal number of samples were mixed according to the PCR product concentration, and the high-throughput sequencing analysis of the soil bacteria 16S rDNA was performed using the Illumina platform Miseq high-throughput sequencing technology (Xu et al., 2019).

### 2.4 Statistical analysis

According to the results of various bioassay indicators, we calculate the allelopathic response index (*RI*) as follows (Williamson and Richardson, 1988):


$$\begin{array}{c} RI=1-\frac{C}{T}(T\geq C)\\ RI=\frac{T}{C}-1(T<C) \end{array}$$

where *T* is the treatment value, and *C* is the control value. *RI* > 0 refers to promotion, and *RI* < 0 to inhibition, where the absolute value is proportional to the intensity of the allelopathy. The integrated allelopathic sensitivity index formula (*SI*) is the arithmetic mean of *RI*.

Regarding the one-way analysis of variance (ANOVA), Tukey’s test for multiple comparisons (*LSD*) and Pearson’s correlation analysis are performed using the SPSS software (IBM, SPSS Version 20.0), with a confidence level of 95%. The redundancy analysis (Canoco 5.0) studies the correlation between soil factors and dominant bacterial phyla. The bioinformatics analysis uses the Vsearch (version 2.4.2) software to classify the high-quality sequence valid tags obtained from quality control according to 97% similarity to out and selected the most abundant sequence in each OTU as the representative OUT sequence. The Chao1 index (S) size estimates the total number of soil bacterial species. Data in the chart are mean ± standard deviation (SD).

## 3 Results

### 3.1 Allelopathic potential of decomposing solution of *E. densa*

**Figure 1:**
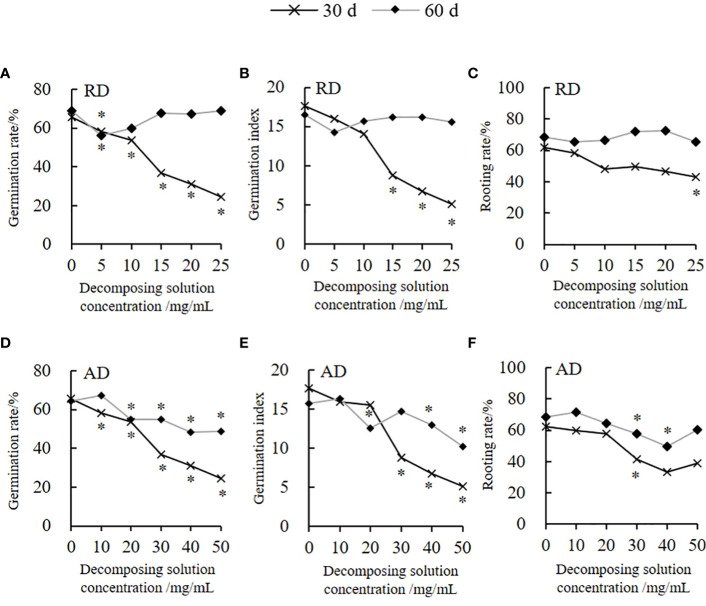
Variation trend of the highland barley seed germination and rooting under *E. densa* decomposing solution treatment. The decomposing part is marked on the upper right side of the Y-axis. “*” indicates the significant difference at the level of 0.05 between different concentrations of decomposing solution. “*” for 30 d and 60 d are marked below and above the broken line, respectively. **(A, D)** *GR*, **(B, E)** *GI*, **(C, F)** *RR*.

The decomposing solution of *Elsholtzia densa* Benth. (*E. densa*) has a specific allelopathic inhibitory effect on the germination of the highland barley seeds (**Figure 1**). Among them, the decomposing solution for 30 days (d) presents the greatest inductive effect. The germination rate (*GR*) and germination index (*GI*) decrease as the decomposing concentration increases; both are lower than the control. Moreover, the decomposing solution has a delayed germination effect. With the prolongation of the decomposing time, the effect of the decomposing solution of *E. densa* on the germination of highland barley seeds weakens. Under the action of 25 mg/mL root decomposing solution (RD) for 30 d, the *GR* of the highland barley is the lowest. Except for 5 mg/mL, there are no significant differences in *GR* and *GI* between RD for 60 d of other treatments and the control (*P* > 0.05) (**Figures 1A, B**). According to the integrated allelopathic sensitivity index (*SI*), the decomposing solution with the shortest decomposing time has the strongest effect of the decomposing solution. The allelopathic effect of RD and the above-ground decomposing solution (AD) for 30 d is 2.56 times and 2.09 times the decomposing solution of the decomposed 60 d, and the *SI* of AD is 2.06 times the RD. The effect of the decomposing solution of *E. densa* on the rooting of highland barley has a time effect (**Figure 1**). The decomposing solution for 30 d has the greatest effect on the rooting of highland barley, and the rooting rate (*RR*) gradually decreases as the decomposing solution’s concentration increases. Under the action of 25 mg/mL RD and 50 mg/mL AD for 30 d, the *RR* of the highland barley is 69.13% and 62.42% of the control group, respectively. There is no significant effect on the *RR* of the highland barley, while the AD presents an increasing-declining-increasing trend as the concentration increases. Furthermore, we find that as the decomposition time prolongs, the inhibitory effect of the decomposing solution on the rooting of the highland barley seeds weakens or even turns into a promoting effect. The allelopathic effects of the RD and AD for 30 d are 4.75 and 1.97 times the decomposing solution for 60 d, respectively, and the *SI* of the AD is 1.52 times that of the RD.

**Figure 2:**
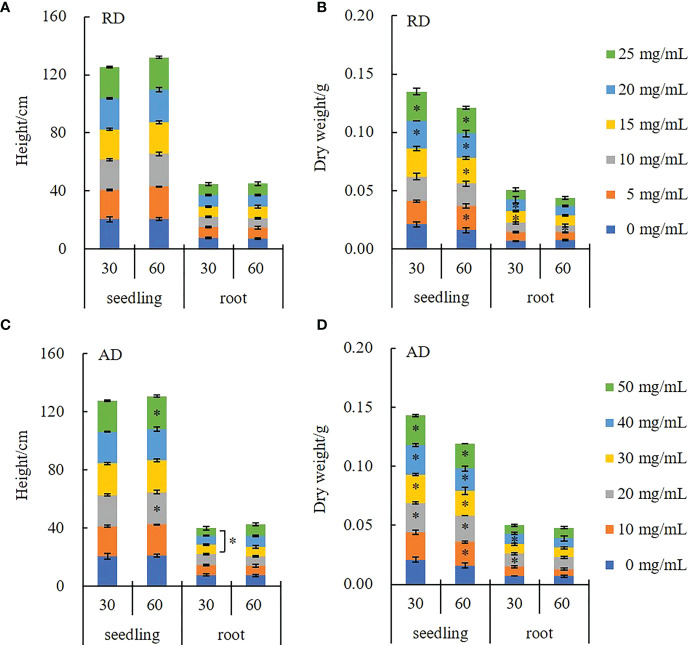
Changes in the growth indexes of highland barley seedlings under the action of *E. densa* decomposing solution. “*” indicates the significant difference at the level of 0.05 between different concentrations of decomposing solution. **(A, C)** height, **(B, D)** dry weight.

**Figure 2** illustrates the effect of the decomposing solution of *E. densa* on the growth of highland barley seedlings. RD has no significant effect on the plant height and root length of the highland barley seedlings (*P* > 0.05). Under the AD action, the highland barley plant height increases in a time-dependent and concentration-dependent manner, while the root length shows a concentration-dependent decrease. In the 50 mg/mL treatment group, the plant height of the decomposing solution for 30 d and 60 d increased by 3.88% and 8.73%, respectively. However, the root length of the 50 mg/mL AD treatment is only 67.87% of that of the control (**Figure 2C**). The effect of the decomposing solution on dry weight has a promoting effect with a time-concentration dual effect. Among them, the highland barley seedlings treated with 50 mg/mL AD for 60 d are dried, with the weight being 1.3 times the control group (**Figure 2D**).

The above study demonstrates that the negative effects of the decomposing process of the stumps of *E. densa* on highland barley are mainly manifested in the effects on seed germination and root growth.

### 3.2 Changes in the characteristics of RBCs of highland barley under the action of a decomposing solution of *E. densa*

**Figure 3:**
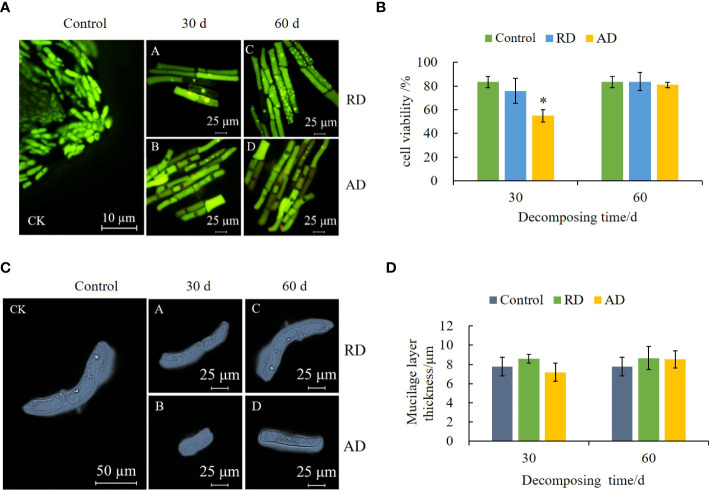
Effects of *E. densa* decomposing solution on cell activity and mucilage layer thickness of RBCs of highland barley. "*" indicates a significant difference at the 0.05 level. **(A)** the fluorescence micrograph under the action of the decomposing solution, green represents living cells, and orange-red represents cells that show the characteristics of cell death. **(B)** the activity of RBCs. **(C)** the micrograph of the RBCs and their mucilage layer of highland barley under the action of the decomposing solution. **(D)** the variation trend of the thickness of the mucilage layer of RBCs.

After AO/EB staining, the root border cells (RBCs) in the control group showed green fluorescence, with a clear cell outline, evident nuclear structure, and bright green fluorescence spots. After treatment with the decomposing solution, the cells’ nuclei are pyknotic, present some apoptosis (**Figure 3A**), and the overall cell viability decreases (**Figure 3B**). The cytotoxicity of the short-term decomposing solution is higher. Compared with the control, the cell viability treated by AD for 30 d decreased by 28.56%. At the same time, the decomposing solution treatment induced the thickening of the mucilage layer of the RBCs (**Figure 3C**), but there was no significant difference from the control (*P* > 0.05) (**Figure 3D**).

### 3.3 Effects of the decomposing process of *E. densa* on soil properties and bacterial community

**Figure 4:**
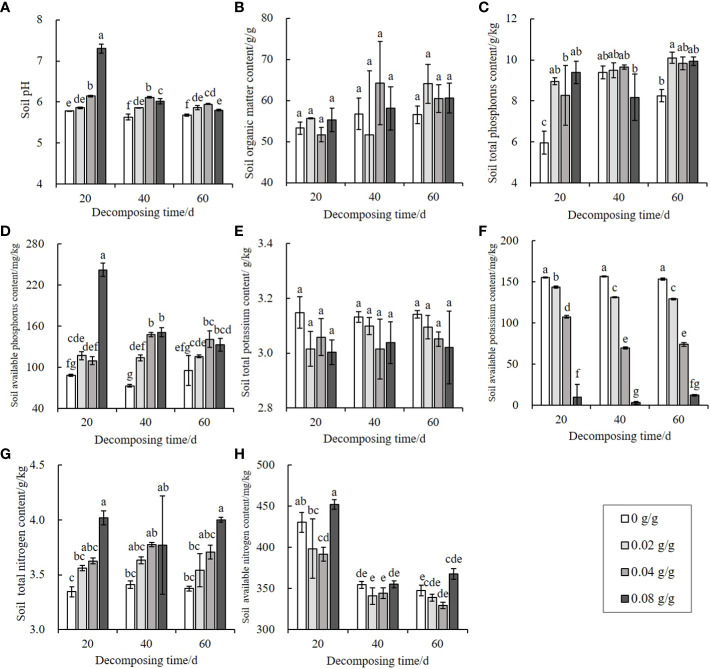
Effects of decomposing *E. densa* on soil pH and nutrients. The color in the column from light to dark indicates that the content of residues of *E. densa* increases. Different decomposition days or concentrations are treated as separate groups. Different lowercase letters indicate the difference significant at the 0.05% level, the same below. **(A)** soil pH, **(B–H)** the content of SOM, TP, AP, TK, AK, TN, and AN on the soil.

The *E. densa* decomposition significantly increases the soil’s pH (*P* < 0.05), with the 0.08 g/g treatment group presenting the largest pH increase after 20 d of decomposition. Over time, the growth amplitudes decrease, but all are higher than the pH of the control group (**Figure 4A**).

In addition to having a minor effect on the soil organic matter (SOM) and total potassium (TK) content (*P* > 0.05) (**Figures 4B, E**), other nutrients are changed to varying degrees during the decomposing process of *E. densa.* Compared with the control, the soiltotal phosphorus (TP) (**Figure 4C**) and available phosphorus (AP) (**Figure 4D**) contents increase, and the changes are the largest when decomposing for 20 d. The difference from the control decreases as the decay time increases but is still higher than the control. The available potassium (AK) content of the treatment group is lower than the control and dose-dependent. The AK content of the 0.08 g/g treatment for 40 d is only 1.92% of the control (**Figure 4F**). The soil total nitrogen (TN) content increases significantly with the increase of the treatment dose (*P* < 0.05), and the maximum treatment dose (0.08 g/g) of decomposing for 20 d has the highest soil TN content, 1.20 times the control (**Figure 4G**). The soil available nitrogen (AN) content first decreases and then increases with the increase of the treatment dose, and decreases with the prolongation of the decomposing time (**Figure 4H**).

**Figure 5:**
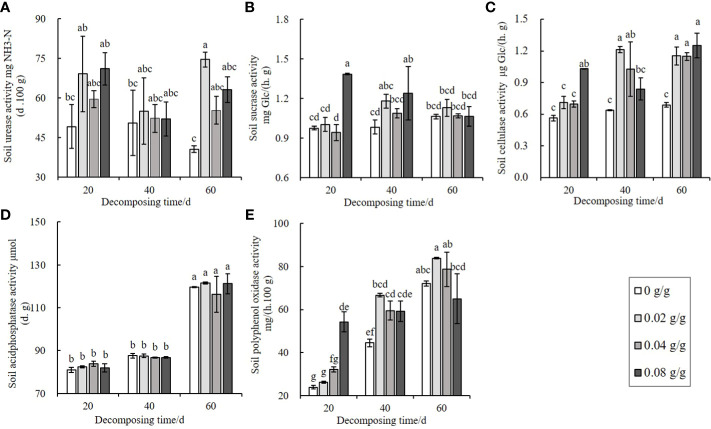
Effects of decomposing of *E. densa* on soil enzyme activities. **(A–E)** the activities of soil urease, soil invertase, soil cellulase, soil acid phosphatase, and soil polyphenol oxidase. Different lowercase letters indicate the difference significant at the 0.05 level.

The decomposing process of *E. densa* increases the activity of extracellular enzymes in soil. The urease activity shows an increasing-decreasing trend with the increase of the treatment dose and reaches a peak at 0.02 g/g (**Figure 5A**). The decomposition time has a minor effect on the urease activity. The changes in sucrase, cellulase, and polyphenol oxidase activities are the same, showing an increasing-decreasing trend with the increase of the treatment dose at 40 d and 60 d, and the activities of the three soil extracellular enzymes increase in a dose-dependent manner at 20 d of decomposing. The activities of sucrase (**Figure 5B**) and polyphenol oxidase (**Figure 5E**) increase the most in the 0.08 g/g treatment group at 20 d. In comparison, the cellulase increases the most at 0.02 g/g at 40 d (**Figure 5C**). The acid phosphatase activity of the 60 d treatment group is significantly higher than the decomposing 20 d and 40 d treatment groups. However, there is no significant difference with the control (**Figure 5D**), indicating that the changes in soil acid phosphatase activity are irrelevant to the decomposition of *E. densa*.

**Figure 6:**
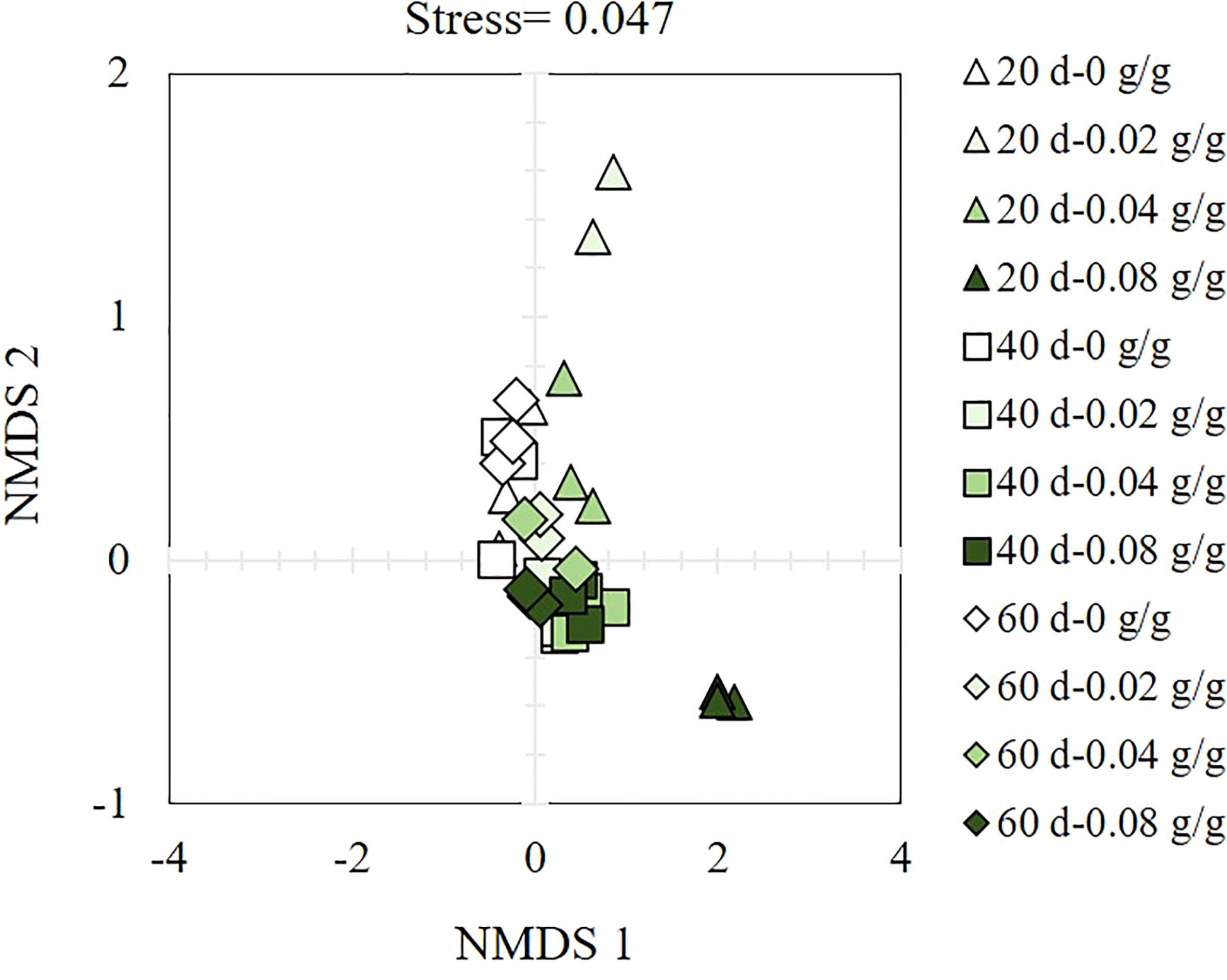
Bacterial β-diversity analysis in the decomposed soil of *E. densa*. The legend reads, “Decomposition time- the content of residues of *E. densa*”, “△” indicates the 20 d treatment group, “□” is the 40 d treatment group, and “◇” is the 60 d treatment group. The green in the graph from light to dark indicates that the content of residues of *E. densa* increases.

The α-diversity index of soil bacteria increases with the prolongation of the decomposing time of *E. densa* (**Table 1**). In different decomposing times, the PD value shows an increasing-decreasing trend with the increase of the residue content of *E. densa* in the soil. Except for the 0.08 g/g treatment decomposed for 20 d, the PD values of the other treatments are higher than the control. The number of observed species shows a decreasing-increasing trend with the prolongation of decomposing time. Among them, the 0.08 g/g treatment group at 20 d has the lowest observed species (1727) and reaches the highest (2485) at 60 d. Shannon-Weiner index and Chao 1 index of the 0.08 g/g treatment at 20 d are lower than the control group. In contrast, the other treatments are higher than the control, indicating that the decomposing process of *E. densa* increases the level of bacterial community diversity. Except that the 20 d decomposing treatment group has a more significant difference within the group, the β-diversity of soil bacteria in the 40 d and 60 d is similar (**Figure 6**).

**Table 1:** Bacterial α-diversity analysis in the decomposed soil of *E. densa*.

Time /d	Residue content g/g	Phylogenetic diversity	Chao 1 index	Observed species	Shannon-Weiner index
20	0	88.54 ± 4.26^ab^	3069.75 ± 132.91^ab^	2369.27 ± 120.39^ab^	9.31 ± 0.17^ab^
20	0.02	90.29 ± 3.6^a^	3031.77 ± 219.18^ab^	2385.2 ± 123.18^ab^	9.44 ± 0.08^a^
20	0.04	90.63 ± 3.27^ab^	3210.79 ± 48.66^ab^	2441.8 ± 62.02^ab^	9.51 ± 0.14^ab^
20	0.08	67.57 ± 0.55^b^	2604.23 ± 22.75^b^	1727 ± 31.42^b^	7.87 ± 0.08^b^
40	0	84.45 ± 1.7^ab^	2996.29 ± 122.86^ab^	2262.67 ± 7.74^ab^	9.05 ± 0.07^ab^
40	0.02	85.89 ± 3.87^ab^	3144.62 ± 81.79^ab^	2293.6 ± 117.67^ab^	9.09 ± 0.27^ab^
40	0.04	88.36 ± 0.99^ab^	3309.81 ± 121.09^a^	2404 ± 60.85^ab^	9.43 ± 0.11^ab^
40	0.08	86.47 ± 3.02^ab^	3164.17 ± 110.98^ab^	2344.77 ± 75.83^ab^	9.38 ± 0.08^a^
60	0	88.98 ± 0.78^ab^	2965.78 ± 21.75^ab^	2395.77 ± 7.62^ab^	9.62 ± 0.07^a^
60	0.02	91.76 ± 2.15^a^	3212.95 ± 10.65^a^	2479.3 ± 47.52^a^	9.54 ± 0.16^a^
60	0.04	92.53 ± 1.46^ab^	3230.53 ± 18.3^a^	2485.95 ± 30.33^ab^	9.53 ± 0.11^ab^
60	0.08	86.34 ± 1^ab^	3189.56 ± 29.53^a^	2349.4 ± 24.8^ab^	9.42 ± 0.04^ab^

Residue content is the content of E. densa residue per gram of soil. 3 replicates per sample. Different lowercase letters indicate the difference significant at the 0.05 level.

**Figure 7:**
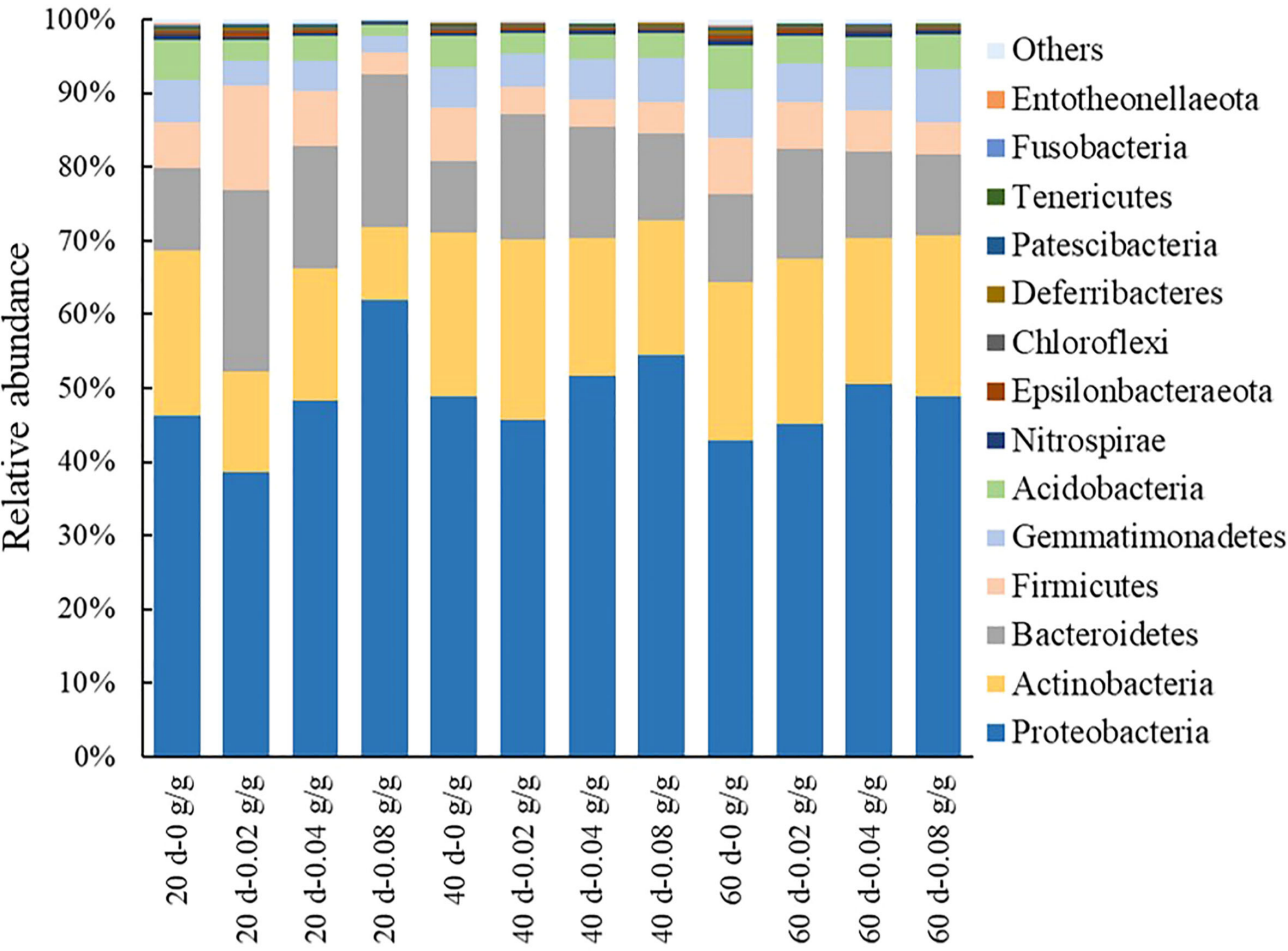
Relative abundance of bacteria at the phyla taxonomic level in the decomposed soil of *E. densa*.

A total of 30 bacterial phyla are detected in the decomposed soil of *E. densa*. During the decomposing process of *E. densa*, the six phyla Proteobacteria, Actinobacteria, Bacteroidetes, Firmicutes, Gemmatimonadetes, and Acidobacteria accounted for the highest proportion (**Figure 7**). The relative abundances of Proteobacteria, Bacteroidetes, Acidobacteria, and Gemmatimonadetes in the decomposed soil increase significantly with the prolongation of the decomposing time. In contrast, the relative abundances of Actinobacteria and Firmicutes decrease significantly. At 20 d and 40 d of decomposition, the relative abundance of Proteobacteria shows a decreasing-increasing trend with the increase of residue content in the soil, while at 60 d of decomposition, it increases first and then decreases with the increase of residue content of *E. densa*, but still higher than the control. With the increased residue content of *E. densa*, the relative abundance of Bacteroidetes increased-decreased. The relative abundance of Firmicutes decreased, and the relative abundance of Acidobacteriaincreased. The relative abundance of Bacteroidetes and Gemmatimonadetes increased with the prolongation of decomposing time.

**Figure 8:**
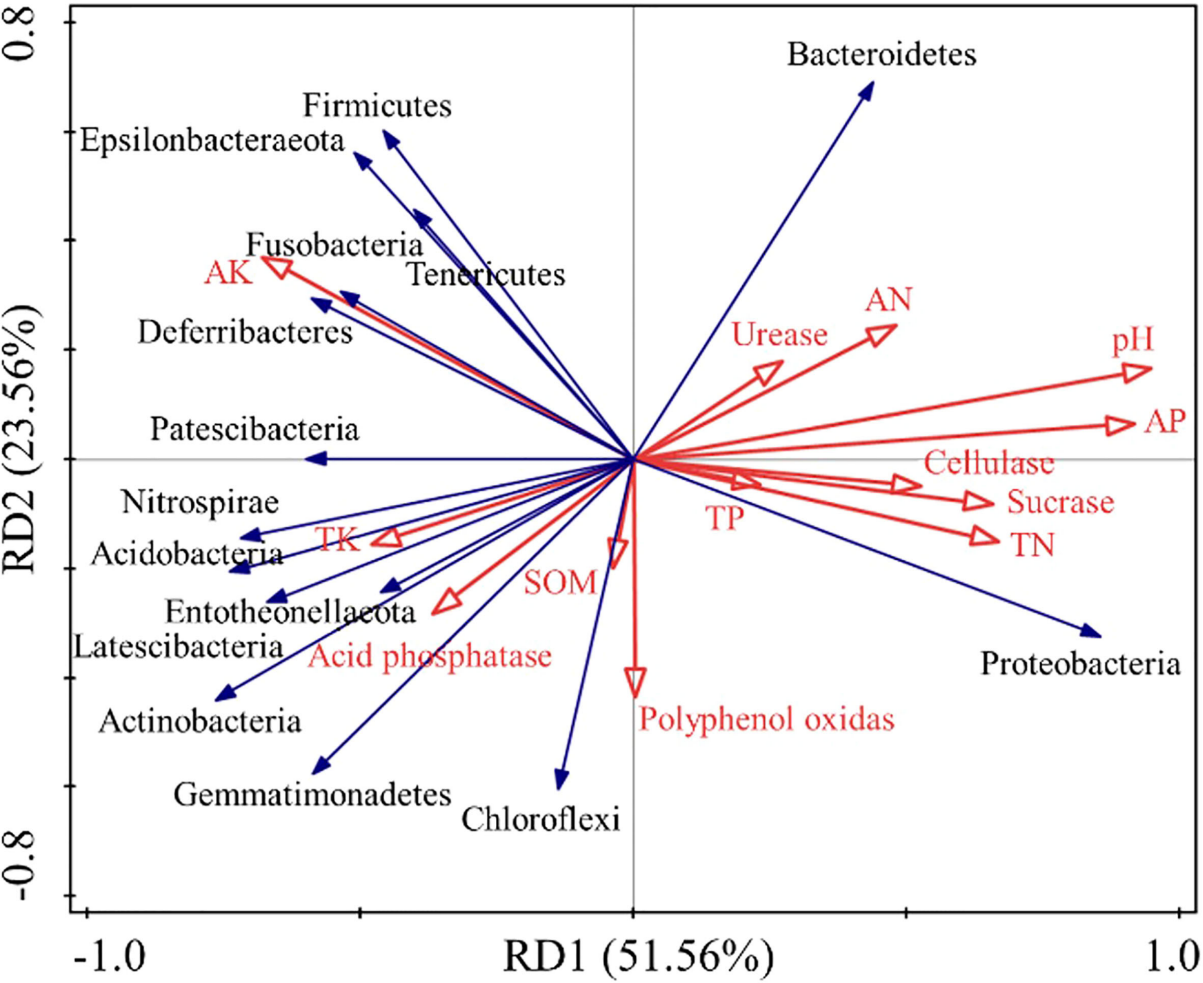
Correlation redundancy analysis between soil bacterial diversity and chemical properties during the decomposing process of *E. densa*. The red arrows represent different environmental factors, and the blue arrows represent different bacteria.

The RDA redundancy analysis (**Figure 8**) highlights that the soil chemical properties during the decomposing process of *E. densa* explain 51.56% of the bacterial community variability, and the two ranking axes explain 75.12% of the variability. Bacteroidetes and Proteobacteria are significantly positively correlated with urease, cellulase, sucrase, TN, AN, AP, and pH. Bacteroidetes and Proteobacteria were negatively correlated with acid phosphatase, AK, and TK. The Actinobacteria are positively correlated with acid phosphatase, polyphenol oxidase, AK, TK, and SOM, while significantly negatively correlated with urease, cellulase, sucrase, TN, AN, AP, and pH. The Firmicutes have a significant positive correlation with AK and TK and a negative correlation with other environmental factors.

## 4 Discussion

### 4.1 The decomposition of *E. densa* has a strong allelopathic effect on the growth of highland barley

In ecosystems, the organization and dynamics of plant communities are controlled by biological processes such as resource competition, promotion, and allelopathy (Zeng, 2008). The active allelochemicals are contained in the organs of many higher plants and play an essential role in plant interactions. During the decomposition of plant residues, allelochemicals are passively released to affect the growth of surrounding plants (Sarheed et al., 2020). Allelochemicals adversely affect the seed germination and growth of neighboring plants by affecting some physiological processes in plants, such as cell division, photosynthesis, respiration, enzyme activity, and cell membrane permeability (Thiébaut et al., 2018; Mushtaq et al., 2019).It can even affect plant community structure and cause the evolution of the ecosystem (Badalamenti et al., 2016). The decomposing solution of the poisonous weed on the QTP, *Steura chamaejasme*, had allelopathic effects on the plant height and biomass of *Onobrychis viciifolia* seedlings. The allelopathy of RD was greater than that of AD. Besides, it also affected the leaf area, leaf cell conductivity, and chlorophyll content of the recipient plants (Zhou et al., 2009). Seed germination is the most sensitive growth stage for plants to external stress (Huang et al., 2020). Our study highlights that decomposing the stubble of the poisonous weed *E. densa* has an apparent inhibitory effect on the seed germination and young root growth of highland barley and exhibits concentration-dependent and delayed effects. Different from *Steura chamaejasme*, the allelopathic inhibitory effect of AD was greater than that of RD made by *E. densa’* stubble. However, this negative effect is mainly reflected in the early decomposition stage, as in the late decomposition stage, the decomposing solution of *E. densa* has a significant promoting effect on the biomass accumulation of highland barley, manifested in the increase of dry weight, especially the dry weight of roots. It should be noted that the allelopathy of *Allium senescens*, a companion species of grassland, was treated with the water extract of *Steura chamaejasme*, the allelopathy of the water extract of *Steura chamaejasme* root was smaller than that of stem and leaf (Liu et al., 2022). Combined with the results of this study, it can be found that the strength and direction of allelopathy are not only related to the site of decomposition and the type of the recipient plant but also related to the stage of decomposition.

### 4.2 The decomposition of *E. densa* interferes with the defense function of highland barley RBCs

RBCs and their secretions form a protective structure, the root extracellular trap (RET), the first line of defense for plants to defend against microorganisms, heavy metals, and other harmful substances invading the root system (Ropitaux et al., 2020). When plants are under allelopathic stress, their RBCs rapidly increase the thickness of the mucilage layer to resist harmful allelochemicals (Ma et al., 2020). To a certain extent, changes in the thickness of the mucilage layer can represent the intensity of external stress. Our study shows that under the action of the decomposed substance of *E. densa*, the activity at the RBCs of the highland barley is reduced, the characteristics of apoptosis are observed, and the thickened mucilage layer is removed. These effects are similar to the decomposing solution effects on seed germination and root length. The shorter the decomposing time, the stronger the cytotoxicity of the decomposing solution. The cytotoxicity of the decomposing solution in the later stage of decomposing weakens or even disappears, and the cytotoxicity of AD is greater than RD. Combined with the experimental phenomenon that root growth is inhibited, it can be speculated that the decomposed substance of *E. densa* interfered with the defense function of RBCs, broke through the biological defense line, and affected the division and elongation activities of root tip cells to block root growth.

### 4.3 The decomposition of *E. densa* changed the soil living environment

*E. densa* is an annual aromatic herb, and the stems and leaves, especially the leaves, contain many volatile substances (Chauhan et al., 2018). The main chemical components in the volatile oil of *E. densa* are α-bisabolol, elements, β-selinene, thymol, and carvacrol, which have antiviral and antibacterial effects (Zhou et al., 2019). Presently, the most reported allelochemical in nature is phenolic compounds, terpenoids, and compounds containing nitrogen atoms (benzoxazines). Under natural conditions, allelopathy is not the effect of a single component but the result of the synergistic action of various allelochemicals (Scavo and Mauromicale, 2021). Allelochemicals produced by the decomposition of plant residues will be chemically transformed into polymers or degraded into small molecules under natural conditions and then lose their allelopathic activity, which is generally considered the self-detoxification function of soil (Rawat et al., 2019). This function is closely related to environmental factors such as soil colloid buffer, soil nutrient content, and soil microflora (Li et al., 2015; El-Darier et al., 2018). Therefore, the main effector allelochemicals of *E.densa* may be released in the early stage of decay but gradually aggregated or decomposed by microorganisms in soil with the prolongation of decay time. The allelopathic effect of *E. densa* residues during decomposition is brief, mainly occurring in the early stage of decomposition. In the later stage of decomposition, with the allelopathic effect of *E. densa* allelochemical weakened, the nutrient elements in the residues were decomposed and released continuously, which promoted the growth of highland barley, such as promoting rooting and increasing dry weight. In the next step, it is necessary to analyze further and verify the main effecting allelochemicals and detect the content changes of the main effecting allelochemical in different decomposition stages for further exploration.

The interaction of plants-microorganisms-soil is the key to realizing plant community exchanges, changing biological diversity, and ecosystem functions (Mueller et al., 2020). As the core driving force of nutrient cycling and energy transformation (Shao et al., 2019), soil microbial communities are extremely sensitive to changes in the external environment, and their quantity and structural composition are affected by the soil environment and the dominant plant groups in the habitat (Yang et al., 2021). Most soil bacteria do not thrive in acidic environments (Li et al., 2022). However, the decomposition of *E. densa* residues increased soil pH, which effectively improved the living environment of soil bacteria and increased the α- diversity of the bacterial community. Meanwhile, the decomposition of *E. densa* stubble changed the bacterial community structure. The β-diversity analysis shows that the bacterial community changes the most in the early 20 d of decomposing, and there is little difference in other decomposing periods. These results are consistent with the results that the decomposition of *E. densa* can inhibit highland barley, mainly in the early stage of decomposition.

Soil properties and pH are stubble factors in determining the distribution of poisonous plants (Li et al., 2014). Actinobacteria were always in a dominant position in the decomposing process of *E. densa.* It can accelerate the decomposition of animal (Araujo et al., 2020) and plant residues in the soil and increase the TN content in the soil with Bacteroidetes (Krishna et al., 2020). In addition, Bacteroidetes also have a phosphorus enrichment effect (Hou et al., 2021), which synergized with Actinobacteria with a phosphorus-dissolving effect (Omotayo et al., 2021) to increase the content of AP in soil. Proteobacteria with increasing relative abundance during decomposition have nitrogen fixation and can adapt to various complex environments (Hou et al., 2019). The larger the proportion of Proteobacteria and Bacteroidetes in soil, the higher the soil fertility (Lu et al., 2020). In the late stage of decomposition, soil pH decreases, and the relative abundance of oligotrophic bacteria, Acidobacteria, which are suitable for growth in an acidic environment, increase and participate in the decomposition of organic matter and the balance of the micro-ecological environment (Wu et al., 2021). The relative abundance of Firmicutes continues to decrease, indicating that the carbon utilization rate continues to decline in the later decomposition stage (Uksa et al., 2017). This corresponds to an increase in the relative abundance of Acidobacteria. Acidobacteria plays an important role in the degradation of plant residues and cellulose (Wang et al., 2016). Acidobacteria and Gemmatimonadetes are more advantageous in an environment with lower water content (Gao et al., 2019). Under the influence of such a complex and constantly changing bacterial influence network, there is no significant change in SOM.

Many hypotheses are used to explain the high competitiveness of invasive plants, such as the rapid growth and reproduction (Ni et al., 2018), high allelopathy, strong adaptability to the heterogeneous environment, and resource assimilation capacity (Gruntman et al., 2013; Wang et al., 2017). Studies have shown that the dominant population of poisonous weeds on the QTP is caused not only by directly suppressing and crowding out other plants through allelopathy but also by changing soil physical and chemical properties and soil microbial community composition by releasing allelochemical (He et al., 2015). Furthermore, the poisonous weed *E. densa* may have a similar ability. Soil enzymes are mainly derived from soil microbial activities, plant root exudates, and enzymes released during the decomposition of animal and plant residues, which play an essential role in soil material cycling and energy transformation (Wu et al., 2020). In our study, hydrolase activities such as urease, sucrase, cellulase, and acid phosphatase which are closely related to soil nitrogen, carbon, and phosphorus cycling (Wang et al., 2020) and redox-related polyphenol oxidase activities (Chen et al., 2020), are increased, indicating that the decomposition of the stubble of *E. densa* can release related enzymes to promote the synthesis of humus components in the soil and the flow and circulation of nutrient elements. We also observe no significant change in TK content but a significant decrease in the AK content. This may be related to the great demand for quick-acting potassium in the decomposition of *E. densa.* According to the resource allocation theory, soil microorganisms may also invest in abundant elements to produce extracellular enzymes to exploit relatively limited elements (Zhou et al., 2020).

Decomposition of *E. densa* stumps stimulates soil microbial activities by changing soil properties and extracellular enzyme activities. It jointly promotes the conversion of soil macromolecules into small molecules and eventually leads to a significant increase in soil phosphorus and nitrogen content. Our hypothesis is further confirmed in the correlation redundancy analysis between soil bacterial diversity and chemical properties. Thus, *E. densa*, which can expand despite land degradation on the QTP, may have adopted similar competitive strategies as invasive plants: takes advantage of the strong allelopathy and resource assimilation ability to transform the surrounding environment to expand its competitive advantage constantly.

## 5 Conclusions

**Figure 9:**
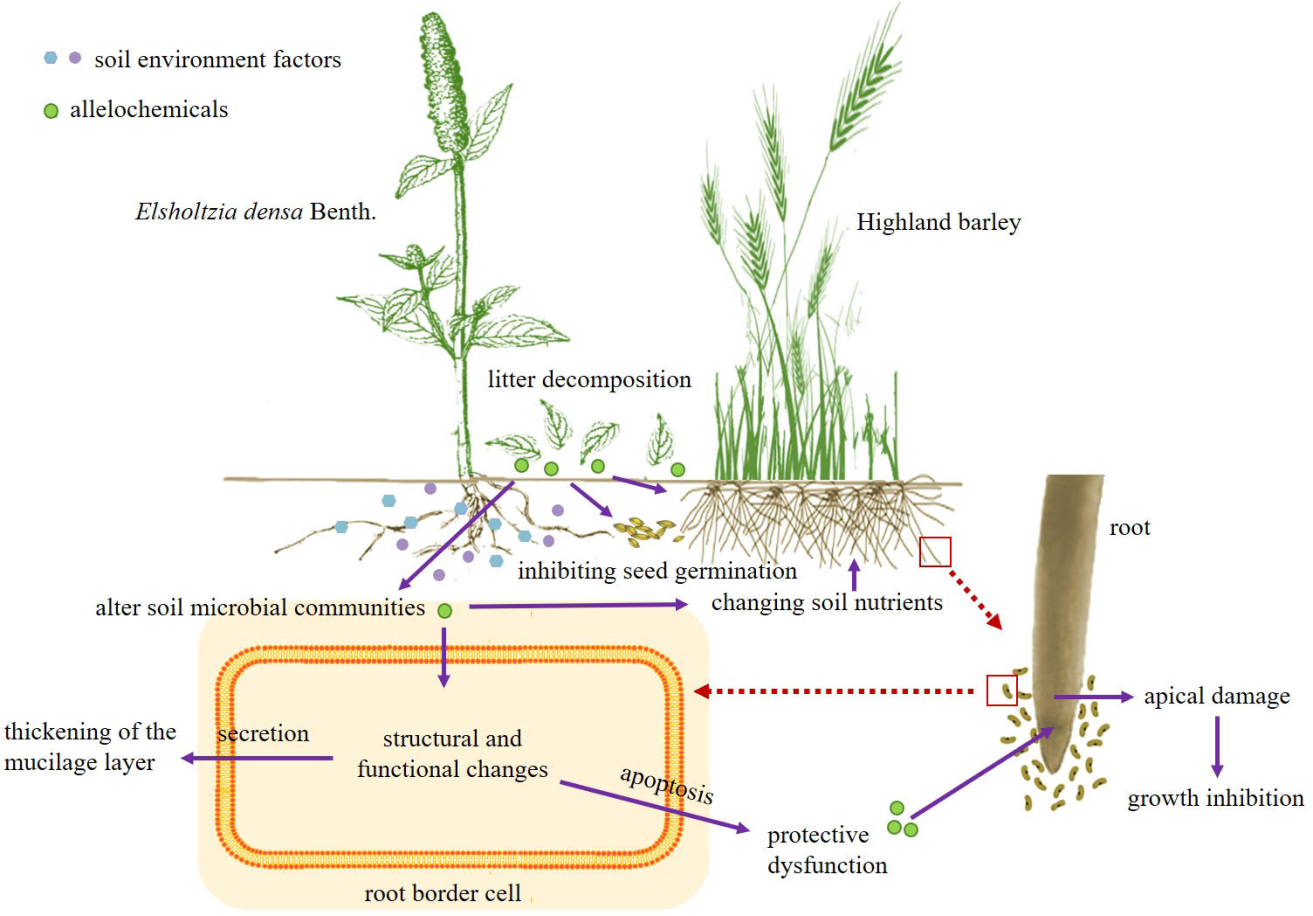
Competitive strategies of *E. densa*.

Our study found that the decomposing solution of *E. densa* had allelopathic effects on the germination and seedling growth of highland barley and interfered with the defense function of the RBCs of highland barley. This effect was related to the time and part of decomposition. The soil environment changed in the decomposition process of *E. densa*. In the early stage of decomposition, soil bacteria varied greatly under different concentrations of *E. densa* residues. In addition, the decomposition process of *E. densa* increased the soil nutrient content, extracellular enzyme activities, and bacterial community diversity. The results showed that the decomposition of *E. densa* residues could stimulate soil microbial activity and promote the flow and circulation of nutrient elements by changing soil properties and extracellular enzyme activities. Therefore, we believe that the competitive strategies adopted by *E. densa* in the farmland ecosystem on the QTP are as follows (**Figure 9**): 1. The release of allelochemicals interferes with the defense function of surrounding plants and directly inhibits the growth and development of surrounding plants. 2. By changing the physical and chemical properties of soil and extracellular enzyme activity, residual plant decomposition can stimulate soil microbial activity, improve soil nutrition status, and create a more suitable soil environment for growth.

## Data availability statement

The data presented in the study are deposited in the NCBI repository, accession number PRJNA914485.

## Author contributions

All authors contributed to the article and approved the submitted version. XZ and YXX: Experiments, analysis and interpretation of data, writing and reviseing the manuscript. DM: Analysis and interpretation of data, writing and reviseing the manuscript. YSX: Experiments, analysis and interpretation of data. YW: analysis and interpretation of data. HZ: Experiments. YNW: analysis and interpretation of data.
